# Detection of TDP‐43 seeds in CSF of presymptomatic and symptomatic genetic FTD/ALS

**DOI:** 10.1002/alz.70989

**Published:** 2025-12-15

**Authors:** Ilaria Linda Dellarole, Vittoria Aprea, Marcella Catania, Claudia Battipaglia, Aurora Romeo, Cristina Villa, Anna Burato, Luigi Celauro, Eleonora Dalla Bella, Nilo Riva, Erika Salvi, Giacomina Rossi, Giuseppe Di Fede, Giuseppe Legname, Julie F H De Houwer, Antonella Alberici, Barbara Borroni, Harro Seelaar, John C van Swieten, Fabio Moda, Paola Caroppo

**Affiliations:** ^1^ SSD Laboratory Medicine‐Laboratory of Clinical Pathology Fondazione IRCCS Istituto Neurologico Carlo Besta Milan Italy; ^2^ Neurology 8 – Dementias and degenerative diseases of CNS Unit Fondazione IRCCS Istituto Neurologico Carlo Besta Milan Italy; ^3^ Laboratory of Prion Biology Department of Neuroscience Scuola Internazionale Superiore Di Studi Avanzati (SISSA) Trieste Italy; ^4^ Neurology 3 – Neuroalgology Unit Fondazione IRCCS Istituto Neurologico Carlo Besta Milan Italy; ^5^ Data Science Center and Computational multi‐Omics of Neurological Disorders (MIND) Lab Fondazione IRCCS Istituto Neurologico Carlo Besta Milan Italy; ^6^ Department of Neurology and Alzheimer Centre Erasmus MC University Medical Centre (Erasmus MC) Rotterdam The Netherlands; ^7^ Department of Continuity of Care and Frailty ASST Spedali Civili Brescia Hospital Brescia Italy; ^8^ Department of Clinical and Experimental Sciences University of Brescia Brescia Italy; ^9^ Molecular Markers Laboratory IRCCS Istituto Centro San Giovanni di Dio Fatebenefratelli Brescia Italy; ^10^ Department of Medical Biotechnology and Translational Medicine University of Milan Milan Italy

**Keywords:** amyotrophic lateral sclerosis, CSF, frontotemporal dementia, SAA, seed amplification assays, TDP‐43

## Abstract

**INTRODUCTION:**

Seed amplification assays (SAAs) have shown promising results in detecting misfolded transactive response (TAR) DNA‐binding protein 43 (TDP‐43) in cerebrospinal fluid (CSF) of genetic frontotemporal dementia (FTD). To date, the use of SAA has yet to be evaluated in presymptomatic individuals.

**METHODS:**

Thirty patients carrying *GRN* or *C9orf72* mutations, 2 microtubule‐associated protein tau (*MAPT*) carriers, 14 presymptomatic subjects, and 27 controls underwent CSF collection. We used SAA for detecting misfolded TDP‐43 (TDP‐43_SAA) and single molecule array (SIMOA) technology for neurofilament light chain (NfL) dosage.

**RESULTS:**

TDP‐43 seeding activity was detected in 67% of TDP‐43‐linked symptomatic patients, with a specificity of 93%. Almost half of presymptomatic subjects tested positive, mostly *GRN* carriers. Interestingly, among TDP‐43_SAA positive presymptomatic individuals, two *GRN* carriers underwent phenoconversion.

**DISCUSSION:**

TDP‐43_SAA can also detect misfolded TDP‐43 in the CSF of presymptomatic individuals. A possible link exists between positive TDP‐43_SAA and conversion to the symptomatic phase.

**Highlights:**

- Seed amplification assay of transactive response (TAR) DNA‐binding protein 43 (TDP‐43_SAA) can detect misfolded TDP‐43 in the cerebrospinal fluid (CSF) of patients with genetic frontotemporal dementia (FTD), linked to *GRN* and *C9orf72* mutations.
- TDP‐43_SAA can detect misfolded TDP‐43 also in the CSF of presymptomatic individuals.
- In both groups, most TDP‐43_SAA positive cases were carriers of *GRN* mutation.
- Two *GRN* carriers that resulted TDP‐43_SAA positive converted to the symptomatic phase of the disease.

## 1 BACKGROUND

Frontotemporal dementias (FTDs) are a group of neurodegenerative diseases (NDs) characterized by high clinical heterogeneity, including progressive language deterioration and behavioral abnormalities, and degeneration of frontotemporal lobes (FTLD). Amyotrophic lateral sclerosis (ALS) is an ND that primarily affects the motor neurons but in about 50% of cases phenotypically overlaps with FTD.^1^, ^2^, ^3^, ^4^ About 20%–25% of FTD cases are caused by mutations occurring primarily in three genes: *MAPT* (encoding microtubule‐associated protein tau), *GRN* (encoding progranulin), and *C9orf72*. Patients with *C9orf72* expansion may present with isolated FTD, ALS, or both. In contrast, patients carrying *GRN* mutations are more frequently associated with a phenotype of behavioral FTD (bvFTD) or primary progressive aphasia (PPA), while ALS is not typically considered part of the phenotypic spectrum associated with *GRN* mutations.^5^, ^6^ From a neuropathological point of view, both *GRN* mutations and *C9orf72* repeat expansion lead to FLTD‐TDP which is characterized by loss of nuclear transactive response (TAR) DNA‐binding protein 43 (TDP‐43) localization and aggregation in neuronal cytoplasm, dendrites, axons and, less frequently, in glial cells such as oligodendrocytes. *GRN* mutations typically lead to FTLD‐TDP type A, while *C9orf72* hexanucleotide repeat expansion leads to FLTD‐TDP type B associated with dipeptide‐repeat protein inclusions.^7^, ^8^, ^9^, ^10^ ALS also pathologically overlaps with FTD with most of the patients presenting TDP‐43 pathology, suggesting a *continuum* between these diseases.^1^, ^2^, ^3^, ^4^, ^11^ Neuropathological differences, as well as the involvement of different brain regions and potential genetic and environmental modifiers, may account for the phenotypical heterogeneity in these disorders. To date, there are no diagnostic markers to detect TDP‐43 proteinopathy in vivo, and the confirmation of the underlying TDP‐43 pathology remains a *post mortem* diagnosis. Indeed, antibody‐based detection of TDP‐43, either the full‐length protein or its post‐translationally modified isoforms, in plasma/serum and CSF has not yielded consistent results.^12^, ^13^ The limitations of direct plasma analysis have recently been overcome through the use of the extracellular vesicles (EVs). A recent study by Chatterjee et al. demonstrated successful detection of elevated TDP‐43 levels, quantified using the SIMOA assay, in plasma EVs derived from patients with behavioral FTD due to TDP‐43 pathology and ALS, relative to controls.^14^ Compared to the methods described above, seed amplification assays (SAAs) enable the ultrasensitive detection of pathological aggregates of misfolded proteins in neurodegenerative proteinopathies. These techniques are currently used in the diagnostic workup of prion diseases on the PrP protein^15^ and synucleinopathies^16^, ^17^ where they have shown strong reproducibility across laboratories, along with high sensitivity and specificity. Recently, promising results emerged from the application of SAA to cerebrospinal fluid (CSF) and olfactory mucosa (OM) samples of genetic TDP‐43 proteinopathies (TDP‐43_SAA).^18^, ^19^ The work of Scialò et al. showed the ability of SAA to amplify TDP‐43 aggregates in CSF samples of patients with *GRN* and *C9orf72* mutations with a total sensitivity of 94% and a specificity of 85%, suggesting its potential as a diagnostic tool.^19^

To date, TDP‐43_SAA has never been applied in a cohort of presymptomatic individuals to test the possibility of detecting pathological changes years before the onset of clinical symptoms. Here, we applied TDP‐43_SAA to a cohort of individuals carrying *GRN* and *C9orf72* mutations to confirm its ability to detect TDP‐43 pathology in vivo. Since TDP‐43 pathology can currently only be confirmed *post mortem*, we decided to analyze CSF exclusively from patients carrying TDP‐43‐related genetic mutations, in whom the presence of TDP‐43 pathology is highly probable. This approach overcomes the pathological uncertainty behind clinical presentation, which is typically observed in sporadic cases.^20^ For the first time, we also evaluated whether TDP‐43_SAA could identify TDP‐43 seeding activity in presymptomatic carriers. Additionally, we measured CSF neurofilament light chain (NfL) levels using single molecule array (SIMOA) technology to explore a potential correlation between (1) NfL, as a marker of neurodegeneration and disease progression, and (2) TDP‐43 seeding activity.

## 2 METHODS

### 2.1 Cohort description

A total of 73 subjects, comprising 32 patients with genetic FTD, FTD‐ALS, or ALS due to *GRN* mutations (*n* = 11), *C9orf72* expansion (*n* = 19) and microtubule‐associated protein tau (*MAPT*) mutations (*n* = 2), 14 presymptomatic carriers (6 *GRN* and 8 *C9orf72*), and 27 cognitively healthy controls (CTRLs) (neuropathy = 10, major depression = 3, subjective cognitive complaint (SCC) = 11, hereditary spastic paraplegia = 1, and healthy controls = 2) were included (Table 1). All controls were cognitively unimpaired, and CSF biomarkers including p‐Tau, total Tau, and the Aβ42/40 ratio, yielded results within normal ranges in controls with SCC, major depression, and hereditary spastic paraplegia. Alzheimer's disease (AD) biomarkers were not available for patients with neuropathy. All included individuals are Caucasian (either from Italy or Netherlands), except for one African descent. All subjects underwent both CSF TDP‐43_SAA and NfL levels detection via SIMOA.

**TABLE 1 alz70989-tbl-0001:** Demographic, clinical data, and NfL dosages of the study population.

Study population	*GRN* *n* = 11	*MAPT* *n* = 2	*C9orf72* *n* = 19	Total cases *n* = 32	CTRLs *n* = 27	*p*‐value (patients vs CTRLs)
**Demographic features**						
Age at CSF collection, years, mean ± SD (range)	59 ± 6 (45–68)	50 ± 12 (42‐59)	61 ± 7 (43–73)	60 ± 7 (42–73)	63 ± 10 (40–79)	*p* > 0.05 ns
Age at onset, years, mean ± SD	56 ± 7	47 ± 14	60 ± 8	57 ± 9	–	–
Disease duration at CSF collection, months, mean ± SD	20 ± 10	36 ± 16	20 ± 15	22 ± 14	–	–
**Sex (F/M) n**.	3/8	2/0	5/14	10/22	10/17	–
**Clinical phenotype n**.						
Pure ALS	0	0	2	2	–	–
ALS plus cognitive decline	0	0	1	1	–	–
FTD‐ALS	0	0	4	4	–	–
bvFTD	5	1	10	16	–	–
nfvPPA	5	0	1	6	–	–
svPPA	0	1	0	1	–	–
PD	1	0	0	1	–	–
CBS	0	0	1	1	–	–
NfL (pg/mL) median (IQR)	4381 (3215.46–5026.86)	2247.88 (2042–2454)	2360 (1582.21–4436.26)	3423.02 (1870.77–4628.04)	649.76 (397.73–909)	*p* ≤ 0.0001 ****
TDP‐43_SAA (+/tot) n.	8/11	0/2	12/19	20/32	2/27	–

Abbreviations: ALS, amyotrophic lateral sclerosis; bvFTD, behavioral variant of frontotemporal dementia; CBS, corticobasal syndrome; CTRLs, controls; F, female; FTD, frontotemporal dementia; IQR, interquartile range; M, male; NfL, neurofilament light chain; nfvPPA, non‐fluent variant of primary progressive aphasia; PD, Parkinson's disease; SAA, seed amplification assay; SD, standard deviation; svPPA, semantic variant of primary progressive aphasia; TDP‐43, transactive response (TAR) DNA‐binding protein 43.

All patients and CTRLs underwent to a standard clinical assessment following local guidelines with neuropsychological evaluation and neurological examination.

**RESEARCH IN CONTEXT**

1. **Systematic review**: Seed amplification assays (SAAs) have already shown promising results in detecting misfolded proteins in cerebrospinal fluid (CSF) and other easily accessible tissues collected from patients with different neurodegenerative disorders, including prion diseases, and alpha‐synucleinopathies. Limited research activities have been carried out in the field of transactive response (TAR) DNA‐binding protein 43 (TDP‐43) proteinopathies. Interestingly, misfolded TDP‐43 was found in the CSF of patients with genetic frontotemporal dementia (FTD), linked to *GRN* and *C9orf72* mutations. However, the CSF of presymptomatic carriers of TDP‐43 pathology has yet to be evaluated using SAA.
2. **Interpretation**: Our preliminary findings suggest that the detectability of TDP‐43 by SAA may be influenced by genetic background and phenotype. In particular, we found that TDP‐43_SAA can detect TDP‐43 in 67% of patients of our cohort with genetic mutations linked to TDP‐43 proteinopathies, with high specificity especially in *GRN* mutation carriers. Data from presymptomatic individuals (43% of whom tested positive for TDP‐43_SAA) were consistent with those from symptomatic patients. In both groups, most TDP‐43_SAA positive cases were carriers of *GRN* mutation, and two positive presymtomatic *GRN* carriers converted to the symptomatic phase of the disease.
3. **Future directions**: Future research involving larger cohorts of both symptomatic and presymptomatic individuals is urgently needed, particularly in light of recent advancements in experimental therapies targeting genetic forms of FTD. Early detection of the pathology and the ability to monitor disease progression are critical for accurately assessing the efficacy of these treatments.

### 2.2 CSF collection

CSF has been collected by lumbar puncture, following a standard procedure. The samples were centrifuged at 1000 × g for 10 min and finally stored in polypropylene tubes at −80°C. Samples were provided by Fondazione IRCCS Istituto Neurologico Carlo Besta, Milano, Italy, University of Brescia, Italy and Erasmus Medical Centre, Rotterdam, the Netherlands.

The study has been approved by the Ethical Commitee of CET Lombardia 4 (approval obtained on November 24, 2023) and Erasmus MC. All patients included in the study signed a written informed consent for samples collection for research.

### 2.3 TDP‐43_SAA analysis

Recombinant human truncated TDP‐43 protein (HuTDP‐43(263‐414)) was produced as previously described^19^ and is referred to as rec TDP‐43. For TDP‐43_SAA, CSF samples were analyzed in black 96‐well optical flat bottom plates (ThermoScientific). Each well was preloaded with one low‐binding glass bead (3 mm, Sigma) and 85 µL of reaction mix prepared as follows: 40 mM Tris at pH 8, 500 mM GdnHCl (pH 8), 10 uM thioflavin T (ThT), 0.002% of sodium dodecyl sulfate (SDS), and 0.05 mg/mL of rec TDP‐43. All solutions were filtered before use with 22 µm sterile filters.

Fifteen µL of CSF samples were added to each well. Samples were tested in triplicate. The plates were sealed with a sealing film and inserted into a FLUOstar CLARIOSTAR microplate reader (BMG Labtech), subsequently incubated at 42°C and subjected to cycles of shaking (15 s, 100 rpm, double orbital) and incubation (30 min). Aggregation was monitored via ThT fluorescence, with readings taken at 480 ± 10 nm emission, expressed in arbitrary units (AU), every 30 min (30 flashes per well at 450 nm excitation). To be considered positive (TDP‐43_SAA+), at least two out of three replicates were required to cross predetermined thresholds of fluorescence (20000 AU) and time (20 h). Negative patients are indicated as TDP‐43_SAA−.[Table alz70989-tbl-0001]

### 2.4 NfL dosage by SIMOA

CSF NfL measurement was performed by SIMOA technology on the SR‐X Analyzer (Quanterix), using the commercially available kit (Neuro 4‐Plex A) according to the manufacturer's instructions. CSF samples were thawed for 60 min at room temperature (RT), vortexed for 10 s and centrifuged for 5 min at 10,000 × g to remove any impurities before use. CSF samples were diluted 40× to a total volume of 152 µL per well. Two quality controls, provided by the manufacturer, were included in each plate. Calibrators were run in triplicates, while samples and controls were run in duplicates, displaying an intra‐assay variation lower than 20%, as assessed statistically. All samples were anonymized and analyzed blindly.

### 2.5 Statistical analyses

Data were analyzed using R (version 4.1.2) and GraphPad Prism 8.0.1 (GraphPad Software). Statistical analyses were performed both for the clinical groups (patients, presymptomatic individuals, and healthy controls) and according to TDP‐43_SAA status (positive vs negative). Categorical variables (genotype, sex, and geographical origin) were compared across groups using Pearson's *χ* ^2^ or Fisher's exact tests, as appropriate. Continuous variables (age and CSF NfL levels) were tested for normality using the Shapiro–Wilk test. As CSF NfL levels were highly skewed, they were log‐transformed prior to parametric analyses. Between‐group differences were evaluated using Kruskal–Wallis tests, followed by Dunn's post hoc tests with Benjamini–Hochberg correction for multiple comparisons. When only two groups were analyzed, a Mann–Whitney test for non‐parametric data was applied. Associations between NfL levels and age were assessed using Spearman's rank correlation coefficient. To account for potential confounders, multivariable linear regression models were fitted with log‐transformed NfL as the dependent variable and age, sex, origin, and clinical status (control, presymptomatic, patient) as fixed effect. Regression coefficients (*β*) were exponentiated to report percent changes relative to the reference group. Models were rerun using presymptomatic carriers as the reference category to enable direct comparisons between patients and presymptomatic individuals. Receiver operating characteristic (ROC) curve analysis (calculating the area under the curve [AUC] ± standard error [SE]) and its 95% confidence interval (CI) was used to assess the diagnostic accuracy of NfL for the discrimination of patients from controls. Statistical significance is denoted as follows: **** *p*‐value < 0.0001; *** *p*‐value < 0.001; and * *p*‐value < 0.05; *p*‐values below 0.05 were considered statistically significant.

## 3 RESULTS

Demographic data, clinical features, SIMOA‐measured NfL levels and results of TDP‐43_SAA are summarized in Table 1 and Table S1 for patients and controls, while characteristics of presymptomatic carriers are shown in Table 2. Although patients tended to be older than controls and presymptomatic carriers, age at CSF collection did not differ significantly among all study groups (Kruskal–Wallis H = 5.41, p = 0.067). Sex distribution was balanced across groups (Pearson's chi‐squared test, p = 0.524), with males representing 62% of the cohort. Regarding clinical phenotypes, the *C9orf72* patients included 10 bvFTD, 1 nfvPPA, 1 corticobasal syndrome (CBS), 4 FTD‐ALS, 2 pure ALS (1 bulbar and 1 spinal onset), and 1 ALS with cognitive impairment (not meeting criteria for FTD diagnosis), *MAPT* carriers included 1 bvFTD and 1 svFTD, 6 *GRN* mutations carriers were diagnosed with bvFTD, 5 with nfvPPA, and 1 with Parkinson's disease (PD) phenotype associated with prodromal FTD (Table 1, Tables S1 and S2). This patient presented with rigid‐akinetic form of PD, responsive to L‐DOPA treatment, with a positive DaTSCAN, associated with mild cognitive executive impairment and mild behavioral disorders. All patients were diagnosed according to the current diagnostic criteria.^21^, ^22^, ^23^

**TABLE 2:** Demographic data of the presymptomatic cohort.

Presymptomatic carriers	*GRN* *n* = 6	*C9orf72* *n* = 8	Total *n* = 14
**Demographic features**			
Age at CSF collection, years, mean ± SD (range)	56 ± 9 (41–67)	53 ± 12 (39–70)	55 ± 11 (39–70)
**Sex (F/M)**	3/3	4/4	7/7
**SAA_TDP‐43**			
SAA+ *n*. (%)	4 (67)	2 (25)	6 (43)
SAA− *n*. (%)	2 (33)	6 (75)	8 (57)
**NfL (pg/ml) median (IQR)**	520.80 (406.145–1520.60)	488.5 (400.55–691.93)	496.15 (400.18–738.18)

*Note*: TDP‐43_SAA results (+ positive;—negative) and NfL dosages are reported.

Abbreviations: CSF, cerebrospinal fluid; F, female; IQR, interquartile range; M, male; NfL, neurofilament light chain; SAA, seed amplification assay; SD, standard deviation; TDP‐43, transactive response (TAR) DNA‐binding protein 43.

### 3.1 TDP‐43 seeding activity in symptomatic patients

To evaluate the ability of TDP‐43_SAA to detect TDP‐43 proteinopathies, we compared 30 patients with *GRN* and *C9orf72* mutations with the other 29 subjects of the cohort (including the 27 CTRLs and the 2 patients with *MAPT* mutations).

**FIGURE 1:**
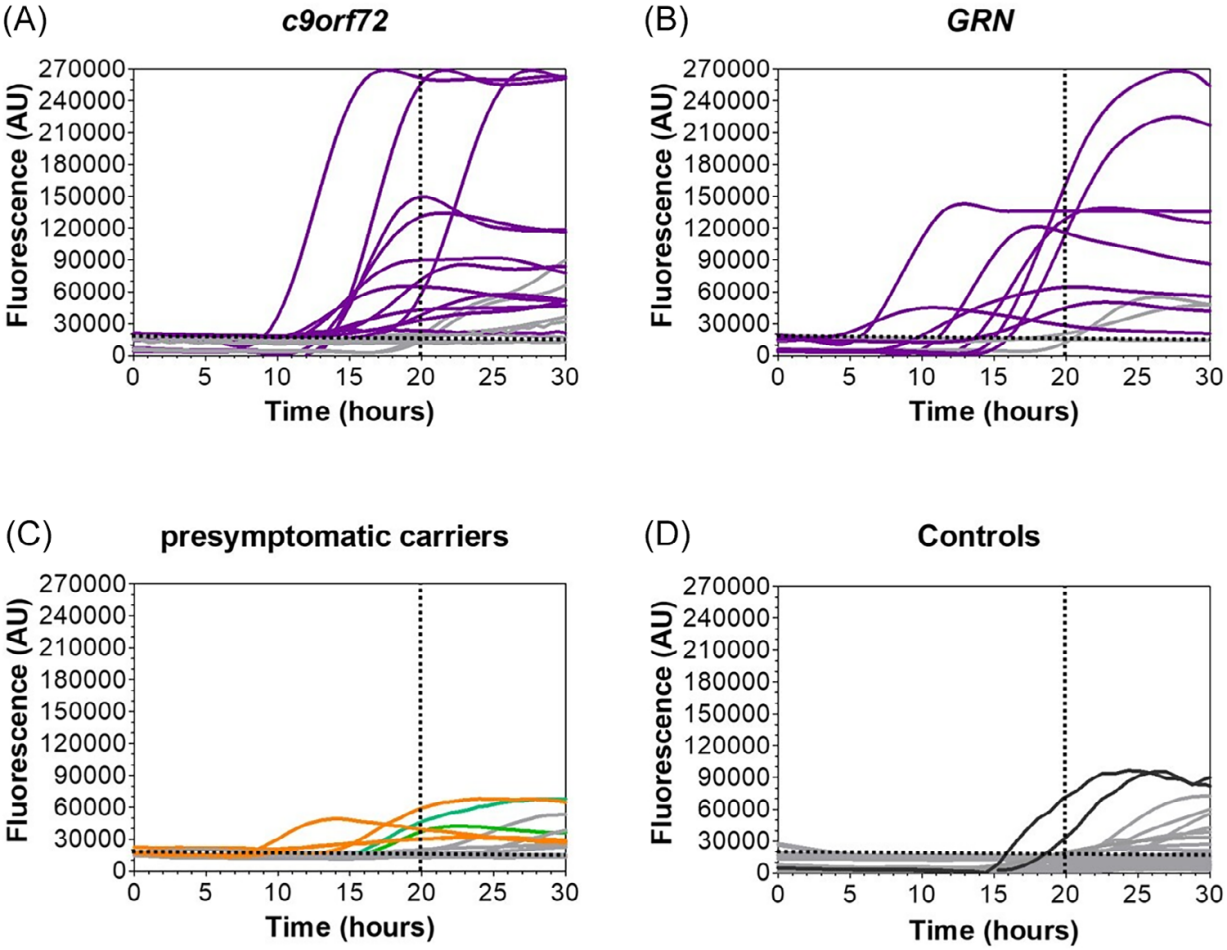
TDP‐43_SAA analyses of CSF from genetic *C9orf72* and *GRN* FTD and FTD‐ALS patients and presymptomatic carriers. Kinetic curves of TDP‐43 seeding activity in CSF from subjects with (A) *C* *9orf72* mutation, (B) *GRN* mutation, (C) presymptomatic carriers, and (D) controls (CTRLs + *MAPT* carriers). (A, B) The kinetic curves are indicated by purple lines for positive CSF from FTD/FTD‐ALS, ALS, and ALS‐FTD patients. (C) Presymptomatic carriers are divided into two groups, depending on the mutation. The green lines indicate *C* *9orf72* carriers, and the orange lines *GRN* carriers. All the samples that tested negative are indicated in gray. (D) The two positive controls are indicated in black, whereas all the samples that resulted negative are indicated in gray. The dotted lines indicate the fluorescence (AU) and time (h) thresholds. ALS, amyotrophic lateral sclerosis; CSF, cerebrospinal fluid; FTD, frontotemporal dementia; MAPT, microtubule‐associated protein tau; SAA, seed amplification assay; TDP‐43, transactive response (TAR) DNA‐binding protein 43.

TDP‐43_SAA was positive in 20 out of 30 samples (67%) from FTD/ALS patients with either *GRN* or *C* *9orf72* mutations (Figure 1). The age at CSF collection for the 10 patients who tested negative was comparable to that of remaining patient population (*p* > 0.05). Similarly, the latency between the onset of symptoms and CSF collection did not differ significantly between these patients and the rest of the cohort (*p* > 0.05).

Considering the specific genotypes, in the group of *C9orf72* carriers (Figure 1A), 12/19 (63%) patients tested positive; specifically, two diagnosed with ALS, 1 with FTD‐ALS and 9 diagnosed with FTD. In the group of *GRN* carriers, 8/11 (73%) FTD patients (Figure 1B) were positive. In the group of CTRLs and *MAPT* patients, 2 out of 29 CSF tested positive (2 CTRLs), yielding a specificity of 93% (Figure 1D).

### 3.2 TDP‐43 seeding activity in presymptomatic carriers

TDP‐43_SAA was positive in 6 out of 14 CSF (43%) from *C9orf72* (2 out of 8 presymptomatic) and *GRN* (4 out of 6 presymptomatic) carriers. The age at CSF collection did not significantly differ between TDP‐43_SAA+ and TDP‐43_SAA− presymptomatc carriers (*p* > 0.05). Remarkably, although not statistically significant, we have found that the fluorescence intensities reached by these samples were lower than those observed in symptomatic patients (Figure **1C**).

### 3.3 CSF NfL levels and TDP‐43_SAA in the study population

**FIGURE 2:**
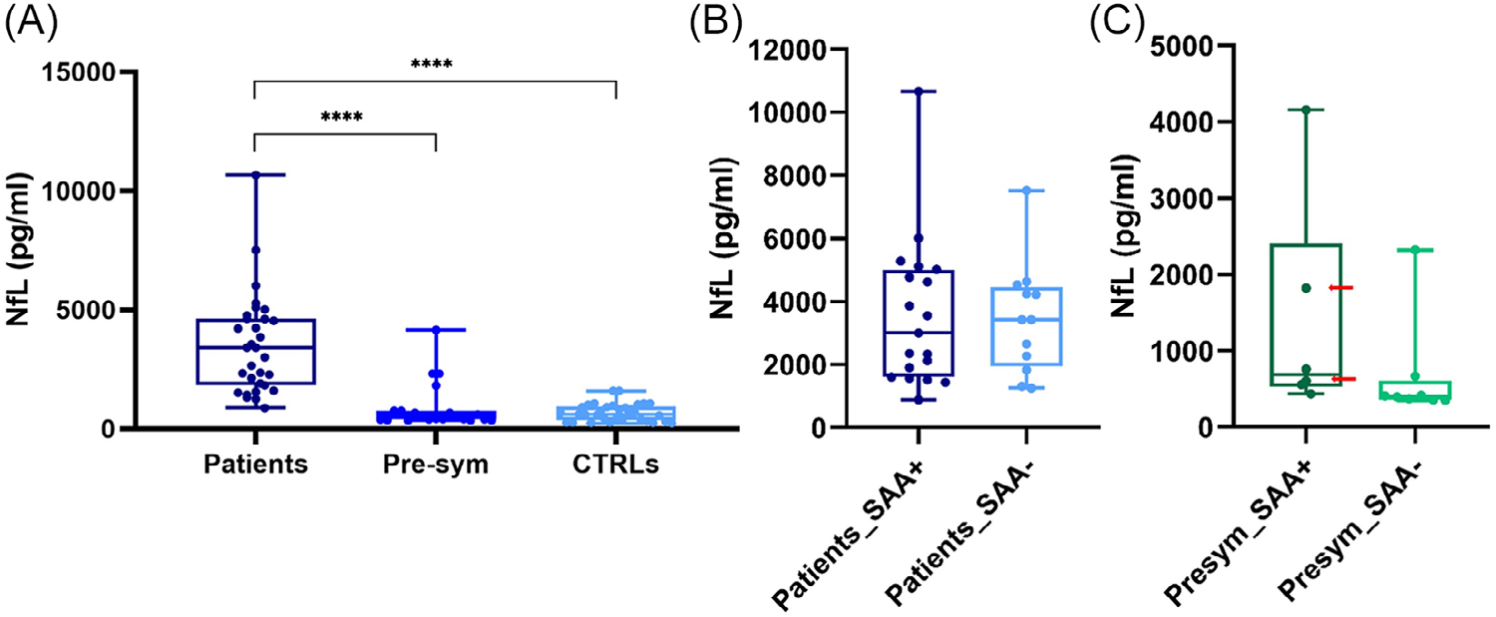
CSF NfL levels by SIMOA in our study population. (A) Statistically significant increase in NfL levels was observed in patients compared to CTRLs and presymptomatic carriers (Pre‐sym) (Kruskal–Wallis test **** *p* < 0.0001). CSF NfL levels and SAA_ TDP‐43 in our study population. (B) NfL levels did not significantly differ between SAA+ and SAA‐ patients (Mann–Whitney test *p* = 0,8889). (C) SAA+ presymptomatic carriers (Presym_SAA+) presented higher NfL levels compared with SAA‐ presymptomatic carriers (Presym_SAA‐) (Mann–Whitney test *p = 0,0426), however the difference was no longer significant after adjusting for age (*p* = 0,097); red arrows show the two *GRN* carriers who underwent phenoconversion. CSF, cerebrospinal fluid; CTRL, control; NfL, neurofilament light chain; SAA, seed amplification assay; SIMOA, single molecule array.

CSF NfL levels were significantly increased in all patients compared to controls and to presymptomatic carriers as well. Kruskal–Wallis (*H* = 43.7, *p* < 0.001) and post hoc Dunn's tests confirmed higher NfL in patients compared with both controls (*p* < 0.001) and presymptomatic carriers (*p* < 0.001), whereas no difference was found between controls and presymptomatic individuals (*p* = 0.84) (Figure 2A). Additionally, NfL showed an excellent discrimination efficiency between patients and controls (AUC value of 0.98 ± 0.01 SE) (Figure S1a). Spearman's correlation showed a weak, non‐significant association between age and NfL levels (*ρ* = 0.125, *p* = 0.30). In multivariable regression adjusting for age, sex, and geographical origin, patient status remained strongly associated with higher log‐transformed NfL (*β* = 1.722, *p* < 0.001; +460% vs controls), while presymptomatic carriers showed a non‐significant trend (+67%, *β* = 0.511, *p* = 0.091). The same results were obtained using presymptomatic carriers as reference, with NfL significantly higher in patients (*β* = 1.211, *p* < 0.001; +236%), and the difference between presymptomatic carriers and controls remained non‐significant (*β* = −0.511, *p* = 0.09). Age had a modest independent effect (+1.9% per year, *β* = 0.019, *p* = 0.023), whereas sex and origin were not significant predictors.

We also compared NfL levels in both patients and presymptomatic carriers according to the genetic background; interestingly, *GRN* mutated subjects, both patients and presymptomatic carriers, presented higher levels of CSF NfL (Table 1) but they did not significantly differ from those of *C9orf72* expansion carriers (Figure S1b,c).

We then compared the results of TDP‐43_SAA and the CSF NfL levels measured by SIMOA (Figure 2). In the patient group, the Mann–Whitney test did not identify differences in CSF NfL levels of patients tested positive (TDP‐43_SAA+) and those of patients tested negative (TDP‐43_SAA−) (Figure 2B). In the presymptomatic carriers subcohort, we found higher levels of CSF NfL in those who tested positive at TDP‐43_SAA compared to presymptomatic carriers who tested negative at TDP‐43_SAA (p < 0.05) (Figure 2C), however this difference was no longer significant after adjusting for age.

## 4 DISCUSSION

The primary aim of our study was to assess the ability of TDP‐43_SAA to detect misfolded TDP‐43 across genetic forms of FTD, and correlate TDP‐43_SAA with different clinical phenotypes, focusing on its potential in presymptomatic individuals.

To this aim, we analyzed the CSF of subjects carrying both *C9orf72* expansion and *GRN* mutations collected at clinical and preclinical disease stages. Considering that the frequency of TDP‐43 pathology increases with age, independently of cognitive status,^24^ we compared age at CSF collection among our study groups (controls, patients, and presymptomatic carriers) and found no significant difference.

Regarding the distinction of *GRN* and *C9orf72* patients from CTRL group and *MAPT* carriers, TDP‐43_SAA reached a sensitivity of 67% and a specificity of 93%.

Interestingly, in the presymptomatic subgroup, 6 out of 14 subjects tested positive for TDP‐43_SAA (Figure 1), corresponding to a sensitivity of 43%. The observed sensitivity in the presymptomatic cohort may reflect the inherent heterogeneity of this group, as presymptomatic carriers could be at different stages of the disease process, with some approaching symptom onset, while others may be further from phenoconversion.

When considering genetic background, we found that most of SAA+ presymptomatic carriers are *GRN* carriers (four out of six (67%) *GRN* tested positive, while only two out of eight (25%) *C9orf72* presymptomatic carriers tested positive). Remarkably, two of *GRN* TDP‐43_SAA+ presymptomatic carriers underwent phenoconversion developing bvFTD after 2 and 3 years of follow‐up, at 62 and 65 years of age, respectively (Figure 2C), while to date, the other carriers remain asymptomatic and continue clinical follow‐up. Therefore, the SAA positivity in the presymptomatic phase of the disease, at least in *GRN* carriers of our cohort, could be more easily detected when the symptomatic phase approaches. In CTRL group, two subjects tested positive at the TDP‐43_SAA. One was a 72‐year‐old woman diagnosed with hereditary spastic paraplegia (HSP) due to *SPG3A* mutation. Notably, HSP is characterized by involvement of the corticospinal motor neurons similarly to ALS, and TDP‐43 pathology has also been found in the HSP subtype SPG6 (caused by mutations in *NIPA1*).^25^ The other subject was a 77‐year‐old man diagnosed with idiopathic neuropathy, a condition in which phosphorylated TDP‐43 aggregates have previously been described in peripheral nerve biopsies.^26^ Given that TDP‐43_SAA detects ongoing pathological TDP‐43 seeding activity, a positive assay result may indicate the presence of coexisting pathology. Moreover, in the absence of neuropathological confirmation, it cannot be excluded that control subjects in whom pathological TDP‐43 seeds were detected, may eventually develop a TDP‐43‐related disorder over time.

Regarding the clinical phenotypes of our patients, 73% of bvFTD and 66% of nfvPPA tested positive at TDP‐43_SAA. Considering the three patients with ALS at onset, two of them tested positive at TDP‐43_SAA while most FTD‐ALS patients tested negative (Table 1 and Tables S1 and S2). However, given the small sample size of subgroups, is not possible to elucidate a clear association of TDP43_SAA positivity with ALS or FTD phenotypes.

In relation to the genotype, in patients we found that most of *GRN* carriers (73%) resulted positive by TDP‐43_SAA (Table 1 and Table S1), similarly to our findings in presymptomatic individuals. According to these observations, differences in genotype or in TDP‐43 filaments between FTLD‐TDP type A and B pathologies could influence the seeding and the propagation of the protein, and consequently the sensitivity of SAA, as already described for other proteinopathies.^27^, ^28^ Consistent with previous findings, NfL levels were significantly higher in patients when compared to presymptomatic carriers and controls.^29^ Although in our sample age showed a modest association with NfL in multivariable models, the mean age did not differ significantly among groups. Moreover, sex and geographical origin were not significantly associated with NfL levels, indicating that these variables did not exert a confounding effect. As already described among familial FTD cases, *GRN* mutation carriers usually exhibit higher NfL levels and faster rates of increase compared to *C9orf72* carriers over time.^30^ In our study, when comparing NfL levels between *GRN* and *C9orf72* patients, we identified a tendency, still non‐significant difference, with *GRN* patients generally presenting higher NfL levels (Figure S1b). Considering the presymptomatic group, NfL levels did not significantly differ from those of controls, consistently with previous findings.^30^ In relation to the genetic background, we found nominally higher NfL levels in *GRN* presymptomatic carriers compared to *C9orf72* carriers, reproducing the trend seen in symptomatic patients (Figure S1c). As we were able to detect TDP‐43 seeding activity in 43% of our presymptomatic carriers, we wondered whether the presence of TDP‐43 aggregates might be associated with the development of neurodegeneration, prior to the onset of clinical symptoms. To test this potential association, we compared NfL levels between TDP‐43_SAA+ and TDP‐43_SAA− presymptomatic carriers, detecting higher NfL levels in TDP‐43_SAA+ presymptomatic subjects; however, in this case, the difference lost significance after adjusting for age (Figure 2C), despite the absence of a statistically significant difference in age between the two groups. Although neuropathological confirmation is not available and our number of presymptomatic carriers is limited, this finding could potentially suggest that neurodegeneration process may have already begun in those presymptomatic individuals who tested positive at TDP‐43_SAA. These subjects presented nominally higher levels of NfL and were older (median age 60 years), compared to the rest of the presymptomatic individuals (median age 53.5 years), which is in line with the evidence that the risk of phenoconversion increases with age.

In the patients, no significant difference in NfL levels was observed between TDP‐43_SAA+ and TDP‐43_SAA− (Figure 2A). Indeed, all patients presented high NfL levels, consistent with previous reports showing that both serum and CSF NfL levels in symptomatic mutation carriers do not change significantly over time and remain elevated.^31^, ^32^

This study has some limitations. First, in the absence of *post mortem* confirmation, we decided to include only patients with TDP‐43 related mutations; while this approach overcame the neuropathological uncertainty associated with sporadic FTD cases, it also limited our sample size. Second, in the *C9orf72* carriers, the presence of poly‐glycine‐proline (poly‐GP) pathology that could contribute to better define the presymptomatic stage of the disease in this genetic form, has not been evaluated. Third, these are cross‐sectional data, therefore the prognostic relevance of our findings in the presymptomatic cohort, particularly in distinguishing between converters and non‐converters, should be validated through serial markers measurements and clinical follow‐up.

## CONFLICT OF INTEREST STATEMENT

Harro Seleelar: Gieskes‐Strijbis Fonds, Alzheimer Nederland (WE.03‐2022‐07); ZonMW [#10510032120003; #10510032120006, # 10510032120002] Bluefield Project to cure FTD; EU‐Horizon project PREDICTFTD (grant agreement n° 101156175) Erasmus Foundation. Nilo Riva: DOD—Department of Defence of the USA; Giovanni Marazzina Foundation; Italian Ministry of Health—PNRR, AriSLA. Barbara Borroni: Medical advisor for Denali, Wave, AviadoBio, Lilly, and UCB. Ilaria Linda Dellarole, Vittoria Aprea, Marcella Catania, Claudia Battipaglia, Aurora Romeo, Cristina Villa, Anna Burato, Luigi Celauro, Eleonora Dalla Bella, Erika Salvi, Giacomina Rossi, Giuseppe Di Fede, Giuseppe Legname_,_ Julie F H De Houwer, Antonella Alberici, John C van Swieten, Fabio Moda and Paola Caroppo reported no disclosures. Author disclosures are available in the supporting information.

## CONSENT STATEMENT

This study was approved by approved by the Ethical Commitee of CET Lombardia 4 (approval obtained on November 24, 2023) and Erasmus MC. All subjects were included according to the study protocol and gave written informed consent.

## Supporting information



Supporting Information

Supporting Information
