# Supplementary material for: Detection of TDP‐43 seeds in CSF of presymptomatic and symptomatic genetic FTD/ALS
Source: Alzheimers Dement. 2025 Dec 15;21(12):e70989. doi: 10.1002/alz.70989 (PMC12706123; doi:10.1002/alz.70989)
Supplement: Supplementary file 1 — Supporting Information [file ALZ-21-e70989-s001.docx]

**Supplementary material**

**Detection of TDP-43 seeds in CSF of presymptomatic and symptomatic genetic FTD/ALS**

| **ID** | **Diagnosis** | **Gene** | **Age at CSF collection** | **Sex** | **TDP-43_SAA** |
| --- | --- | --- | --- | --- | --- |
| c9orf72_1 | CBS | *C9orf72* | 60 | M | + |
| c9orf72_2 | nfPPA | *C9orf72* | 63 | F | + |
| c9orf72_3 | ALS plus cognitive decline | *C9orf72* | 57 | M | + |
| c9orf72_4 | FTD-ALS | *C9orf72* | 61 | M | - |
| c9orf72_5 | FTD-ALS | *C9orf72* | 73 | F | - |
| c9orf72_6 | bvFTD | *C9orf72* | 43 | F | - |
| c9orf72_7 | ALS-spinal | *C9orf72* | 62 | M | - |
| c9orf72_8 | ALS-bulbar | *C9orf72* | 69 | F | + |
| c9orf72_9 | bvFTD | *C9orf72* | 66 | M | - |
| c9orf72_10 | bvFTD | *C9orf72* | 61 | M | + |
| c9orf72_11 | bvFTD | *C9orf72* | 63 | M | - |
| c9orf72_12 | bvFTD | *C9orf72* | 58 | M | + |
| c9orf72_13 | bvFTD | *C9orf72* | 63 | M | + |
| c9orf72_14 | bvFTD | *C9orf72* | 55 | M | + |
| c9orf72_15 | bvFTD | *C9orf72* | 70 | M | + |
| c9orf72_16 | bvFTD | *C9orf72* | 63 | M | + |
| c9orf72_17 | FTD-ALS | *C9orf72* | 72 | M | - |
| c9orf72_18 | bvFTD | *C9orf72* | 52 | F | + |
| c9orf72_19 | FTD-ALS | *C9orf72* | 64 | M | + |
| GRN_1 | nfPPA | *GRN* | 59 | M | + |
| GRN_2 | bvFTD | *GRN* | 61 | F | + |
| GRN_3 | bvFTD | *GRN* | 68 | M | + |
| GRN_4 | nfPPA | *GRN* | 61 | F | + |
| GRN_5 | nfPPA | *GRN* | 45 | M | - |
| GRN_6 | bvFTD | *GRN* | 53 | F | + |
| GRN_7 | PD | *GRN* | 62 | M | + |
| GRN_8 | bvFTD | *GRN* | 63 | M | + |
| GRN_9 | nfPPA | *GRN* | 57 | M | - |
| GRN_10 | bvFTD | *GRN* | 66 | M | - |
| GRN_11 | nfPPA | *GRN* | 60 | M | + |
| MAPT_1 | bvFTD | *MAPT* | 59 | F | - |
| MAPT_2 | svPPA | *MAPT* | 42 | F | - |
| CTRL_1 | SCC | - | 67 | M | - |
| CTRL_2 | SCC | - | 60 | F | - |
| CTRL_3 | Hereditary spastic paraplegia | - | 72 | F | + |
| CTRL_4 | MDD | - | 74 | M | - |
| CTRL_5 | SCC | - | 67 | M | - |
| CTRL_6 | SCC | - | 58 | F | - |
| CTRL_7 | SCC | - | 70 | M | - |
| CTRL_8 | CIDP | - | 59 | F | - |
| CTRL_9 | MDD | - | 55 | M | - |
| CTRL_10 | MDD | - | 79 | M | - |
| CTRL_11 | HC | - | 47 | M | - |
| CTRL_12 | HC | - | 40 | M | - |
| CTRL_13 | SCC | - | 64 | M | - |
| CTRL_14 | SCC | - | 48 | F | - |
| CTRL_15 | SCC | - | 75 | M | - |
| CTRL_16 | SCC | - | 62 | F | - |
| CTRL_17 | SCC | - | 61 | M | - |
| CTRL_18 | SCC | - | 60 | F | - |
| CTRL_19 | CIDP | - | 61 | M | - |
| CTRL_20 | CIDP |  | 51 | F | - |
| CTRL_21 | Idiopatic Neuropathy | - | 77 | M | + |
| CTRL_22 | Polineuropathy in hATTR | *TTR* | 68 | M | - |
| CTRL_23 | MGUS Associated Peripheral Neuropathy | - | 70 | M | - |
| CTRL_24 | Vasculitic neuropathy in Sjögren's syndrome | - | 75 | F | - |
| CTRL_25 | Idiopatic Neuropathy | - | 63 | M | - |
| CTRL_26 | Idiopatic Neuropathy | - | 76 | M | - |
| CTRL_27 | Diabetic polyneuropathy | - | 49 | F | - |

**Supplementary Table 1. TDP-43_SAA results according to genotype and phenotype.**  Abbreviations: M = male; F = female; bvFTD = behavioural variant of frontotemporal dementia; nfvPPA = non fluent variant of primary progressive aphasia; svPPA = semantic variant of primary progressive aphasia; PD = Parkinson's disease; CBS= corticobasal syndrome; SCC = subjective cognitive complain; MDD = major depressive disorder; CIDP = Chronic Inflammatory Demyelinating Polyneuropathy; hATTR= Hereditary transthyretin amyloidosis; MGUS = Monoclonal gammopathy of undetermined significance; HC = healthy control. + positive; - negative.

| **Clinical Phenotype n.** | **Gene** | **TDP-43 SAA +**  **n.** | **TDP-43 SAA -**  **n.** | **NfL (pg/ml) median (IQR)** |
| --- | --- | --- | --- | --- |
| Pure ALS n=2 | *C9orf72* | 1 | 1 | 4941,84 (3650,88-6232,80) |
| ALS plus cognitive decline n=1 | *C9orf72* | 1 | 0 | 5282,34 |
| FTD-ALS n= 4 | *C9orf72* | 1 | 3 | 3835,89 (2957,79-4344,40) |
| bvFTD n=10 | *C9orf72* | 7 | 3 | 2015,45 (1539,03-3149,89) |
| nfvPPA n=1 | *C9orf72* | 1 | 0 | 1430 |
| CBS n=1 | *C9orf72* | 1 | 0 | 4623,97 |
| bvFTD n=5 | *GRN* | 4 | 1 | 4774,46 (4222,42-5110,87 |
| nfvPPA n=5 | *GRN* | 3 | 2 | 4200 (4369,70-5084.82) |
| PD n=1 | *GRN* | 1 | 0 | 883,67 |
| svPPA n=1 | *MAPT* | 0 | 1 | 2659,80 |
| bvFTD n=1 | *MAPT* | 0 | 1 | 1835,96 |

**Supplementary Table 2. TDP-43_SAA results and NfL levels according to genotype and phenotype.** Abbreviations: bvFTD = behavioural variant of frontotemporal dementia; nfvPPA = non fluent variant of primary progressive aphasia; svPPA = semantic variant of primary progressive aphasia; PD = Parkinson's disease; CBS= corticobasal syndrome


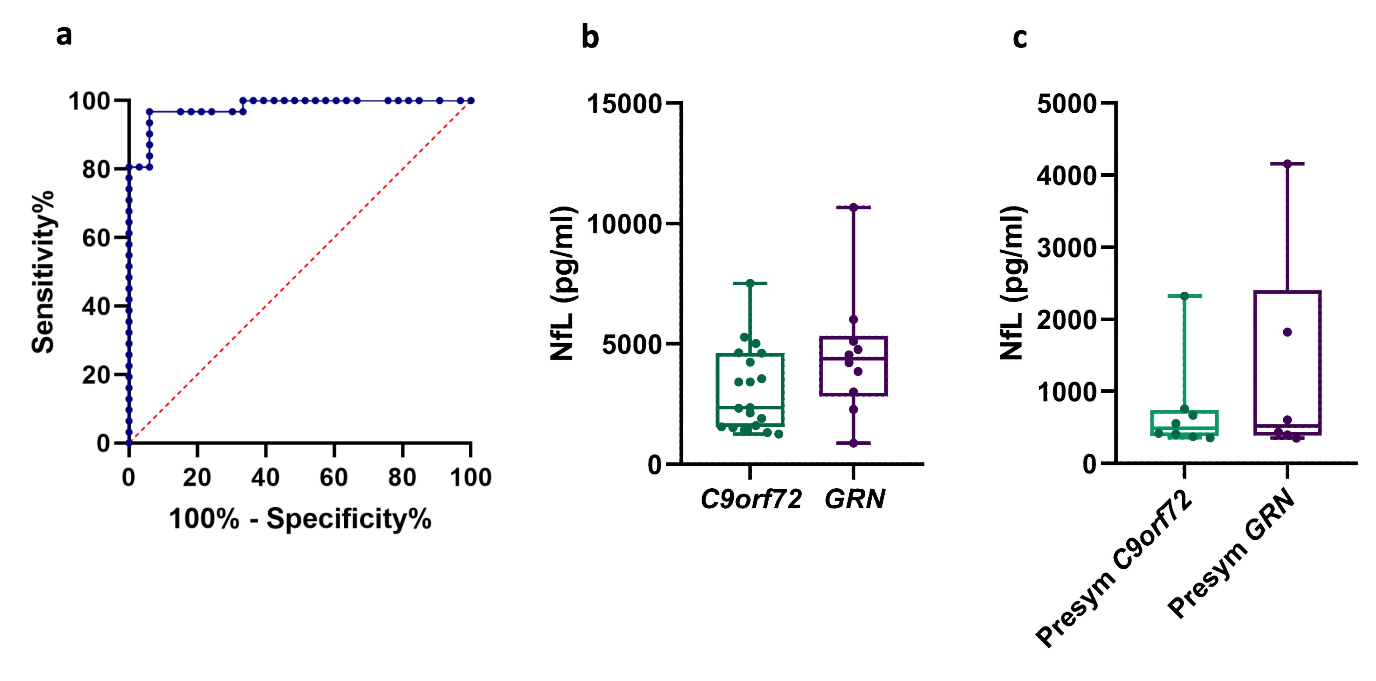


**Supplementary Figure 1. a** Receiver operating characteristic (ROC) curve analysis of CSF NfL from patients and controls. The diagnostic accuracy of CSF NfL was assessed for the discrimination of patients from controls. AUC value of 0.98 ± 0.01 SE indicated excellent diagnostic accuracy. **b** CSF NfL levels did not significantly differ between C9orf72 and GRN patients (Mann-Whiteny test p= 0.1378), as well as between C9orf72 and GRN presymptomatic carriers (**c**) (Mann-Whitney test p= 0.8518), however GRN mutated subjects presented nominally higher levels of CSF NfL compared to C9orf72 expansion carriers.
